# Identifying the competencies of doctors in China

**DOI:** 10.1186/s12909-015-0495-y

**Published:** 2015-11-25

**Authors:** Li Zhao, Tao Sun, Bao-Zhi Sun, Yu-Hong Zhao, John Norcini, Lincoln Chen

**Affiliations:** 1 School of Public Health, Harbin Medical University, Harbin, China; 2 Beijing Tian Tan Hospital, Capital Medical University, Beijing, China; 3 China Medical University, 92 North Second Road, Heping District, Shenyang, China; 4 Foundation for Advancement of International Medical Education and Research, Philadelphia, USA; 5 China Medical Board, Cambridge, MA USA

## Abstract

**Background:**

China adopted a Flexnerian model as its medical institutions developed over the recent past but the political, social, and economic environment has changed significantly since then. This has generated the need for educational reform, which in other countries, has largely been driven by competencies-oriented models such as those developed in Canada, and the United States. Our study sought to establish the competencies model, relevant to China, which will support educational reform efforts.

**Methods:**

Data was collected using a cross-sectional survey of 1776 doctors from seven provinces in China. The surveys were translated and adapted from the Occupational Information Network General Work Activity questionnaire (O*NET-GWA) and Work Style questionnaire (O*NET-WS) developed under the auspices of the US Department of Labor. Exploratory factor analysis and confirmatory factor analysis ascertained the latent dimensions of the questionnaires, as well as the factor structures of the competencies model for the Chinese doctors.

**Results:**

In exploratory factor analysis, the questionnaires were able to account for 64.25 % of total variance. All responses had high internal consistency and reliability. In confirmatory factor analysis, the loadings of six constructs were between 0.53 ~ 0.89 and were significant, Construct reliability (CR) were between 0.79 ~ 0.93 respectively. The results showed good convergent validity. The resultant models fit the data well (GFI was 0.92, RMSEA was 0.07) and the six-factor competencies framework for Chinese doctors emerged.

**Conclusions:**

The Chinese doctors’ competencies framework includes six elements: (a) technical procedural skills; (b) diagnosis and management; (c) teamwork and administration; (d) communication; (e) professional behavior; and (f) professional values. These findings are relevant to China, consistent with its current situation, and similar to those developed in other countries.

**Electronic supplementary material:**

The online version of this article (doi:10.1186/s12909-015-0495-y) contains supplementary material, which is available to authorized users.

## Background

There has been a growing focus on outcomes-based medical education over the past two decades, driven by the desire to ensure that physicians have the right knowledge, skills, and attitudes to serve their patients and communities [1, 2]. To support this growing trend, several groups around the world have identified the competencies that the doctor are required to have. The “global minimum essential requirements (GMER)” were sponsored by the China Medical Board of New York (CMB) in 1999 and implemented in a number of Chinese medical schools [3]. The USA’s Accreditation Council for Graduate Medical Education (ACGME) Outcome Project (1999) introduced six domains of clinical competencies and the Royal College of Physicians and Surgeons of Canada (RCPSC) developed CanMEDS as a framework for physician competencies organized around seven roles [4]. Other frameworks have been developed by The General Medical Council of UK [5], the Indiana University School of Medicine (IUSM) [6], and researchers in Taiwan [7]. Although there are some differences among them, these general medical frameworks have a considerable amount in common.

Application of these general frameworks is underway in several countries. For example, the GMC developed a version of Good Medical Practice for each specialty in 2002 and the ACGME has drafted milestones for many specialties [5.8]. However, across countries there is considerable diversity in both the delivery of healthcare and the nature of medical education, which might result in variability in the competencies as well [8]. Given these differences, it is preferable to generate local competencies rather adapting or adopting those that already exist in other countries [9]. The purpose of this study is to identify the competencies necessary for good medical practice in China.

Doctors are the backbone of the Chinese health care system because they play a crucial role in patient management, disease prevention, and health promotion. As a result, the quality of medical care can be improved by making changes in medical education system. A first step in this direction is the development of competencies that might help to drive reforms in medical education.

Several methods for developing competencies are available including theoretical analysis, behavioral event interview, the Delphi method [10, 11], the expert-novice approach [12, 13], and work analysis [14, 15]. For example, the GMER competencies were created by the leadership of the International Institute on Medical Education [3]. The CanMEDS project developed the competencies by consulting experts and healthcare organizations, conducting a systematic review of the literature, and undertaking a Delphi process [4]. The ACGME Outcome Project convened 33 experts who decided the competencies of doctors [16], These are qualitative methods.

The Occupational Information Network (O*NET) questionnaire (O*NET) was developed under the auspices of the US Department of Labor as a tool for work analysis [17]. The U.S. Department of Labor sponsors an online, freely available O*NET database which reports the results of various job analyses and makes available the questionnaires it uses. O*NET methods have been applied successfully to a wide range of jobs, including those in healthcare and teaching. Importantly, research has shown that the O*NET questionnaires have good applicability to jobs in healthcare and they have been useful in building physician competencies models in the US [17–22].

Among the cluster of O*NET questionnaires, the General Work Activity (GWA) and Work Style (WS) questionnaire were suitable tools to assess competencies [23]. This study reports the results of administering the O*NET-GWA and O*NET-WS questionnaires to doctors from seven provinces in China [18]. The American Psychological Association defined general work activity as “an aggregation of similar job activities/behaviors that underline the accomplishment of major work functions” [24]. The O*NET-GWA questionnaire included four domains: information input, mental processes, work output and interacting with others [25]. It can be considered as the element that above the “iceberg” of competencies [26, 27]. Meanwhile, Work Style was defined as “work- and job-related personal characteristics” [28]. The O*NET -WS questionnaire included two domains: decision-making work styles and interpersonal work styles [29]. It can be considered as the element that beneath the “iceberg” of competencies model [26, 27]. This is a quantitative method [23] and most previous surveys about competencies applied qualitative methods [10–15]. These data were submitted for analysis to produce competencies model of doctors that could be used in the reform of Chinese medical education.

## Methods

### Questionnaires

To collect the opinions of doctors on the competencies that doctors should have after the 3 years of residency training or with the equal experience, we used the O*NET -GWA (Additional File 1: GWA quetionnaire) and the O*NET-WS questionnaires (Additional File 2: WS questionnaire). The O*NET-GWA questionnaire consisted of 41 items such as “How important is getting information to the performance of your current job?” The O*NET-WS questionnaire consisted of 16 items such as “How important is persistence to the performance of your current job?” Responses of the O*NET-GWA and O*NET-WS questionnaire were all captured on 5-point Likert scales where 1 is “Not important” and 5 is “Extremely important”.

The questionnaire was translated to Chinese by a panel of health professionals including experienced doctors, nurses, and a clinical psychologist working at a teaching hospital. It was pretested on 20 doctors, who were asked to comment on the acceptability and clarity of the items and the scale as a whole. The final translated items used for data collection were generated through consensus on the wording, clarity and cultural equivalence of items.

### Participants

There were 23,170 hospitals with 2,138,836 doctors in China by the end of 2010 according to the Statistics Year Book 2011 [30]. We recruited doctors with the help of the North China Center for Medical Education Development (NCC) [31], which is a collaboration of 18 medical institutions representing most of China (i.e., well beyond northern China). The NCC collaborative institutions administered the questionnaires in their local provinces.

We performed stratified sampling to ensure those surveyed were similar to the population of doctors throughout China. Data were collected from 7 provinces which covered 7 different geographic regions of China [32]: Shandong Province (East China), Guangxi Province (South China), Shanxi Province (Central China), Hubei Province (North China), Liaoning Province (northwest China), Sichuan Province (Southwest China), and Xinjiang Province (Northeast China).

Sampling was stratified at the level of institutions to match the percentage of all certified physicians employed in the medical institutions in China according to China Health Statistics Yearbook 2011 [30]. In each province, we surveyed 1 medical college affiliated hospital with 60 doctors; 1 provincial hospital with 60 doctors; 2 municipal hospitals with 35 doctors each; 1 district hospital with 12 doctors; 2 rural hospitals with 19 doctors each; 2 community health service centers (CHSC) with 10 doctors each; 2 rural clinics with 10 doctors each. In total, 280 doctors in 11 medical establishments were surveyed in each province and 1960 doctors in 77 hospitals were surveyed throughout China.

### Data collection

Seven trained surveyors collected data from May to September 2012. The surveyors received training together by the same person to ensure they understood the questionnaire and how the data needed to be coded. They administered the questionnaire in person to each participant. The surveyor spent about 5 min explaining the purpose of the study and then the participants were given 20 min to complete the questionnaire independently. The responses of the participants were anonymous and the results remained confidential. In addition to the questionnaire, the demographic and occupational characteristics of the participants were gathered.

Of the 1960 questionnaires, 63 were removed from analysis because the doctors indicated that they did not wish to participate, 30 were removed because they lacked demographic information, 70 were removed because more than 10 questions (20 % of the questions) were unanswered [33–36], and 21 were removed because the participant marked the same answer to all questions. This left 1776 questionnaires (90.61 %) for analysis. If less than 20 % questions were unanswered, the missed data would be replaced with means [33–36].

### Data analysis

There were two stages of analysis. In the first stage, exploratory factor analysis (EFA) was conducted to establish the factor structure of the scale. In the second stage, confirmatory factor analysis (CFA) was conducted to verify the factor structure and to ascertain the competencies of Chinese doctors.

The sample size requirements for these analyses were a 1:10 to 1:15 ratio of questions to participants [25]. Total questions on the O*NET-GWA and O*NET-WS surveys were 57 so between 570 and 855 participants were adequate for the analysis, and the number of participants far exceeded these values. To perform exploratory factor analysis and confirmatory factor analysis, the participants were randomly divided into two groups of approximately 50 % each for analysis.

In exploratory factor analysis, after deducting the overlap between each of the 57 items and its related domain, factor loadings of more than 0.60 were considered satisfactory [25]. Items with factor loading of less than 0.6, or with cross factor loadings greater than 0.35 were removed from further analysis. The Kaiser-Meyer-Olkin- Kriterium (KMO) statistic and Bartlett’s spherical check were calculated to check for sample suitability for the factor analysis There are two ways which determined the number of factors for consideration. Firstly, it is Kaiser's eigenvalue-greater-than-one rule (K1 or Kaiser criterion) [34]. We compute the eigenvalues for the correlation matrix and determine the number of the eigenvalues greater than 1. This number is the number of factors included in the model. Secondly, it is Cattell's scree plot [35]. We compute the eigenvalues for the correlation matrix, and then plot the values. By examining the graph, we determine the last substantial drop in the magnitude of eigenvalues. The number of plotted points before the last drop is the number of factors included in the model. The factors were recalculated after items were removed from initial exploratory factor analysis. Cronbach’s α coefficient was calculated as an estimate of the reliability of the questionnaire. An alpha of 0.7 to 0.9 was considered good [36].

In confirmatory factor analysis, the selection of the best fitting model was based on several fit indices. For acceptable model fit, chi-square should be low, the Comparative Fit Index (CFI) should be higher than 0.90, and Root Mean Square Error of Approximation (RMSEA) should be lower than 0.08 [37]. Maximum likelihood was used to estimate parameters in these analyses.

The data were analyzed using IBM SPSS® version 17.0 and AMOS® version 20.0 (SPSS Inc., Chicago, IL, USA) for Windows®. A *P*-value of < 0.05 was considered to be statistically significant.

### Ethical approval

The Bioethics Advisory Commission of Harbin Medical University approved the protocol. The completed questionnaires did not contain any identifying information about the individual subjects. Each participant gave written consent. Participation in the study was totally voluntary, participants were paid, and they had the option of declining to answer specific questions or to leave the entire questionnaire blank. All data were kept confidential and data protection was observed at all stages of the study.

## Results

### Characteristics of the doctors and the questionnaires

The average age of the doctors was 39.45 years (SD = 8.20), with 919 (51.7 %) male doctors and 857 (48.3 %) female doctors, and the ratio was similar to the male to female ratio of the doctors in China (1.34:1) according to China Health Statistics Yearbook 2011. The sample includes 401 trainees, 572 attending physicians, 463 associate professors and 340 chairs of the department. Of the participants, 519 were general internists, 425 were surgeons, 408 were gynaecologists, 340 were pediatricians and 84 were from others clinical departments. There were 1460 urban doctors and 316 rural doctors. The demographic characteristics of the participants are shown in Table 1. Frequencies and means for each item were shown in Table 2.

**Table 1:** Demographic characteristics

Characteristic		N (%)
Gender	Male	919(51.7)
Age (mean, SD)		39.45 ± 8.20
Education	MB (Bachelor of Medicine)	873(49.2)
	MM (Master of Medicine)	643(36.2)
	MD (Doctor of Medicine)	260 (14.6)
Title	Resident doctor	401(22.6)
	Attending physician	572(32.2)
	Associate professor	463(26.1)
	Chairman of department	340(19.1)
Specialty	General internists	519(29.2)
	Surgeons	425(23.9)
	Gynecologist	408(23.0)
	Pediatricians	340(19.1)
	Others	84(4.7)
Area	Urban doctors (provincial hospital, college affiliated hospitals and municipal hospital)	1460(82.2)
	Rural doctors (district general hospital, rural hospital, rural clinic and Community health service center)	316(17.8)
Total		1776(100.0)

**Table 2:** Frequencies and means for each item

Item	N (%)					Mean, SD
	1	2	3	4	5	
WS1	19(1.1)	88(5.0)	580 (32.7)	967(54.4)	122(6.9)	3.61 ± 0.73
WS2	4(0.2)	48(2.7)	503(28.3)	1025(57.7)	196(11.0)	3.77 ± 0.68
WS3	8 (0.5)	47(2.6)	496(27.9)	1010(56.9)	215(12.1)	3.78 ± 0.70
WS4	58(3.3)	168(9.5)	636(35.8)	762(42.9)	152(8.6)	3.44 ± 0.90
WS5	1(0.1)	37(2.1)	465(26.2)	1010(56.9)	263(14.8)	3.84 ± 0.69
WS6	7(0.4)	91(5.1)	652(36.7)	886(49.9)	140(7.9)	3.60 ± 0.72
WS7	41(2.3)	137(7.7)	652(36.7)	797(44.9)	149(8.4)	3.49 ± 0.84
WS8	5(0.3)	55(3.1)	493(27.8)	978(55.1)	245 (13.8)	3.79 ± 0.72
WS9	9(0.5)	41(2.3)	449(25.3)	997(56.1)	280(15.8)	3.84 ± 0.72
WS10	7(0.4)	52(2.9)	515 (29.0)	954(53.7)	248(14.0)	3.78 ± 0.73
WS11	3(0.2)	32(1.8)	484(27.3)	952(53.6)	305(17.2)	3.86 ± 0.72
WS12	2(0.1)	45 (2.5)	432(24.3)	968(54.5)	329(18.6)	3.89 ± 0.73
WS13	5(0.3)	44(2.5)	479(27.0)	912(51.4)	336(19)	3.86 ± 0.75
WS14	10(0.6)	50(2.8)	495(27.9)	920(51.8)	301(16.9)	3.82 ± 0.76
WS15	22(1.2)	104(5.9)	611(34.4)	859(48.4)	180(10.2)	3.60 ± 0.80
WS16	8()0.5	47(2.6)	456(25.7)	967(54.5)	298(16.8)	3.84 ± 0.74
WA1	6(0.3)	66(3.7)	496(27.9)	1041(58.6)	167(9.4)	3.73 ± 0.69
WA2	10(0.6)	73(4.1)	562(31.6)	957(53.9)	174(9.8)	3.68 ± 0.73
WA3	38(2.1)	145(8.2)	608(34.3)	806(45.4)	179(10.1)	3.53 ± 0.86
WA4	45(2.5)	192(10.8)	615(34.6)	800(45)	124(7)	3.43 ± 0.87
WA5	72(4.1)	243(13.7)	726(40.9)	663(37.4)	72(4.1)	3.24 ± 0.88
WA6	25(1.4)	172(9.7)	687(28.7)	773(43.5)	119(6.7)	3.44 ± 0.81
WA7	24(1.4)	154(8.7)	698(34.3)	79.8(45)	102(5.7)	3.45 ± 0.79
WA8	44(2.5)	168(9.5)	664(37.4)	780(44)	120(6.8)	3.43 ± 0.85
WA9	38(21)	170(9.6)	628(35.4)	802(45.2)	138(7.8)	3.47 ± 0.85
WA10	13(0.7)	90(5.1)	517(29.1)	940(52.9)	216(12.2)	3.71 ± 0.77
WA11	30(1.7)	115(6.5)	609(34.3)	830(46.8)	192(10.8)	3.58 ± 0.83
WA12	6(0.3)	46(2.6)	494(27.8)	974(54.8)	256(14.4)	3.80 ± 0.72
WA13	17(1)	104(5.9)	61734.7()	864(48.7)	174(9.8)	3.60 ± 0.78
WA14	37(2.1)	104(5.9)	690(38.9)	820(46.2)	125(7)	3.50 ± 0.80
WA15	29(1.6)	103(5.8)	654(36.8)	826(46.5)	164(9.2)	3.56 ± 0.80
WA16	139(7.8)	264(14.9)	738(41.5)	560(31.6)	75(4.2)	3.09 ± 0.97
WA17	468(26.4)	377(21.3)	483(27.2)	392(22.1)	56(3.2)	2.54 ± 1.19
WA18	284(16)	361(20.3)	572(32.2)	496(27.9)	63(3.5)	2.83 ± 1.11
WA19	27(1.5)	153(8.6)	583(32.9)	809(45.6)	204(11.5)	3.57 ± 0.86
WA20	107(22.9)	348(19.6)	544(30.6)	415(23.4)	62(3.5)	2.65 ± 1.17
WA21	445(25.1)	446(25.2)	468(26.4)	368(20.6)	49(2.8)	2.51 ± 1.15
WA22	436(24.5)	436(24.6)	463(26.1)	392(22.1)	49(2.8)	2.54 ± 1.16
WA23	325(18.3)	465(26.2)	498(28)	432(24.4)	56(3.2)	2.68 ± 1.12
WA24	56(3.2)	254(14.3)	617(34.7)	693(39)	157(8.8)	3.36 ± 0.94
WA25	201(11.3)	388(21.8)	585(32.9)	524(29.5)	78(4.4)	2.94 ± 1.07
WA26	12(0.7)	101(5.7)	569(32)	902(50.8)	192(10.8)	3.65 ± 0.77
WA27	73(4.1)	187(10.5)	654(36.8)	738(41.6)	124(7)	3.37 ± 0.91
WA28	17(1)	123(6.9)	616(34.7)	852(48)	168(9.5)	3.58 ± 0.79
WA29	24(1.4)	163(9.2)	711(40)	751(42.3)	127(7.2)	3.45 ± 0.81
WA30	67(3.8)	218(12.3)	702(39.5)	683(38.4)	106(6)	3.31 ± 0.85
WA31	32(1.8)	119(6.7)	591(33.3)	822(46.3)	212(12)	3.41 ± 0.84
WA32	39(2.23)	181(10.2)	693(39.1)	743(41.8)	120(6.8)	3.41 ± 0.84
WA33	11(0.6)	98(5.5)	577(32.5)	883(49.8)	207(11.7)	3.66 ± 0.78
WA34	27(1.5)	102(5.7)	578(32.5)	841(47.4)	228(12.9)	3.64 ± 0.83
WA35	38(2.1)	100(5.6)	682(38.4)	782(44.1)	174(9.8)	3.54 ± 0.83
WA36	49(2.8)	128(7.2)	634(35.7)	790(44.5)	175(9.9)	3.51 ± 0.87
WA37	36(2)	170(9.6)	668(37.6)	767(43.2)	135(7.6)	3.45 ± 0.84
WA38	33(1.9)	202(11.4)	711(40)	743(41.9)	87(4.9)	3.37 ± 0.82
WA39	170(9.6)	296(16.7)	658(37)	560(31.6)	92(5.2)	3.06 ± 1.03
WA40	183(10.3)	297(16.8)	652(36.7)	562(31.6)	82(4.6)	3.03 ± 1.04
WA41	206(11.6)	303(17.1)	653(36.8)	544(30.5)	70(3.9)	2.98 ± 1.05

### Exploratory factor analysis

The data were randomly divided into two groups of approximately 50 % using SPSS. One group contained 917 participants and it was used for exploratory factor analysis based on principal components analysis (PCA). The underlying dimensions were assumed to be correlated with each other and promax rotation was applied to relax the orthogonal constraint to allow for correlated factors [25]. The Kaiser-Meyer-Olkin-Kriterium (KMO) statistic was 0.97 and Bartlett’s spherical check was *χ*2 = 38417.52(df = 1596)and *P* < 0.001. Together these indicated that the study data were suitable for factor analysis.

For the factor analysis of the O*NET-WS questionnaire, items WS5 (Cooperation), WS6 (Concern for Others), and WS15 (Innovation) had loadings >0.35 on more than one construct and they were removed from analysis. WS7 (Social Orientation) had a factor loading of less than 0.6, and it was also removed. Twelve items were left and the analysis yielded two factors. Given the content of the items, the factors were named (a) professional behavior; and (b) professional values (Table 3).

**Table 3:** Exploratory factor analysis results of the O*NET-WS scales

Items	Factors	
	Professional behavior	Professional values
WS12 Attention to Detail	0.79	
WS11 Dependability	0.76	
WS14 Independence	0.72	
WS13 Integrity	0.71	
WS9 Stress Tolerance	0.68	
WS10 Adaptability/Flexibility	0.67	
WS16 Analytical Thinking	0.66	
WS8 Self-Control	0.65	
WS5 Cooperation	0.61	0.43
WS6 Concern for Others	0.52	0.52
WS1 Achievement/Effort		0.75
WS4 Leadership		0.74
WS3 Initiative		0.70
WS2 Persistence		0.66
WS7 Social Orientation		0.57
WS15 Innovation	0.45	0.53
Variance	32.88 %	23.52 %

For the factor analysis of the O*NET-GWA questionnaire, items WA5 (Estimating the Quantifiable Characteristics of Products, Events, or Information), WA31 (Resolving Conflicts and Negotiating with Others), and WA39 (Performing Administrative Activities) had loadings greater than 0.35 on more than one factor and they were removed from further analysis. WA1 (Getting Information), WA11 (Thinking Creatively), WA12 (Updating and Using Relevant Knowledge), WA14 (Scheduling Work and Activities), WA16 (Performing General Physical Activities), WA19 (Working with Computers), WA24 (Documenting/Recording Information),WA30 (Selling or Influencing Others), WA32 (Performing for or Working Directly with the Public), WA38 (Providing Consultation and Advice to Others) and WA40 (Staffing Organizational Units) had factor loadings less than 0.6, and they were also removed. 27 items were left for analysis and 4 factors were identified. Given the content of the items, the factors were named (a) technical procedural skills; (b) diagnosis and management; (c) teamwork and administration; and (d) communication (Table 4).

**Table 4:** Exploratory factor analysis results of the O*NET-GWA scales

Items	Factors				
	Diagnosis and management	Technical procedure	Teamwork and administration	Communication	5
WA 7 Evaluating Information to Determine Compliance with Standards	0.71				
WA 9 Analyzing Data or Information	0.70				
WA 8 Processing Information	0.69				
WA 3 Monitoring Processes, Materials, or Surroundings	0.68				
WA 4 Inspecting Equipment, Structures, or Materials	0.67				
WA 10 Making Decisions and Solving Problems	0.67				
WA 6 Judging the Qualities of Objects, Services, or People	0.64				
WA 5 Estimating the Quantifiable Characteristics of Products, Events, or Information	0.64	0.44			
WA 2 Identifying Objects, Actions, and Events	0.63				
WA 13 Developing Objectives and Strategies	0.60				
WA 15 Organizing, Planning, and Prioritizing Work	0.60				
WA 12 Updating and Using Relevant Knowledge	0.57				
WA1 Getting Information	0.57				
WA14 Scheduling Work and Activities	0.56				
WA 11 Thinking Creatively	0.55				
WA 21 Drafting, Laying Out, and Specifying Technical Devices, Parts, and Equipment		0.86			
WA22 Repairing and Maintaining Mechanical Equipment		0.86			
WA 23 Repairing and Maintaining Electronic Equipment		0.81			
WA 20 Operating Vehicles, Mechanized Devices, or Equipment		0.80			
WA 17 Handling and Moving Objects		0.79			
WA 18 Controlling Machines and Processes		0.77			
WA 25 Interpreting the Meaning of Information for Others		0.65			
WA 41 Monitoring and Controlling Resources		0.61			
WA 16 Performing General Physical Activities		0.57			
WA 40 Staffing Organizational Units		0.54			
WA39 Performing Administrative Activities		0.50	0.47		
WA 36 Guiding, Directing, and Motivating Subordinates			0.78		
WA 37 Coaching and Developing Others			0.74		
WA 34 Developing and Building Teams			0.73		
WA 35 Training and Teaching Others			0.70		
WA 33 Coordinating the Work and Activities of Others			0.64		
WA 38 Providing Consultation and Advice to Others			0.55		
WA 28 Establishing and Maintaining Interpersonal Relationships				0.73	
WA 27 Communicating with People Outside the Organization				0.72	
WA 29 Assisting and Caring for Others				0.66	
WA 26 Communicating with Supervisors, Peers, or Subordinates				0.64	
WA 30 Selling or Influencing Others				0.59	
WA 32 Performing for or Working Directly with the Public				0.54	
WA 31 Resolving Conflicts and Negotiating with Others			0.41	0.51	
WA19 Working with Computers					0.56
WA24 Documenting/Recording Information					0.42
Variance	18.95 %	17.96 %	12.02 %	11.62 %	2.87 %

The initial percentage of variance accounted for by Professional behavior, Professional values, Diagnosis and management, Technical procedure, Teamwork and administration, and communication were 32.88 %, 23.52 %, 18.95 %, 17.96 %, 12.02 %, and 11.62 % respectively.

After deleting the items, we performed the exploratory factor analysis again. The Mean (SD) for final factor scores and percentage of variance accounted for by each factor are shown in Table 5. The included items in each factor were the same as the first exploratory factor analysis after deleting the items. The percentage of variance accounted for by professional behavior and professional values of the O*NET-WS questionnaire were 37.06 % and 22.86 % respectively. The percentage of variance accounted for by technical procedural skills, diagnosis and management, teamwork and administration and communication of the O*NET-GWA questionnaire were 21.06 %, 19.90 %, 14.36 % and 11.36 % respectively. The Mean (SD) for professional behavior, professional values, technical procedural skills, diagnosis and management, teamwork and administration and communication were 30.78(4.54), 14.62(2.38), 21.73(7.46), 35.31(6.19), 21.39(3.84) and 21.39(3.84) respectively.

**Table 5:** The Mean (SD) for final factor scores and percentage of variance accounted for by each factor

	Mean (SD)	Variance
Professional behavior	30.78(4.54)	37.06 %
Professional values	14.62(2.38)	22.86 %
Diagnosis and management	35.31(6.19)	19.90 %
Technical procedure	21.73(7.46)	21.06 %
Teamwork and administration	21.39(3.84)	14.36 %
Communication	14.02(2.81)	11.36 %

The final Cronbach’s alpha of the two questionnaires were 0.90 and 0.94, satisfying the requirement of being greater than 0.7. The minimum corrected-item-total correlation was 0.45, which was greater than what is recommended and the entire model explained 64.25 % of the variance.

### Confirmatory factor analysis

**Fig. 1:**
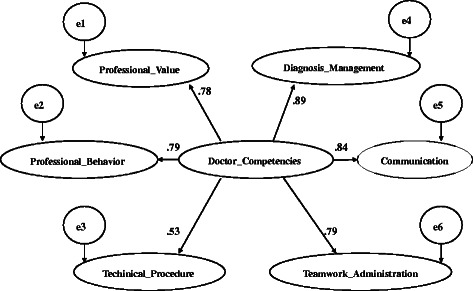
Results of second-order confirmatory factor analyses. Arrows showed causal paths. All paths were significant standardized path coefficients. Ovals signify latent variables (i.e. the constructs of doctors’ competencies of GWA and WS items)

The other half of the data was used for confirmatory factor analysis (CFA). The construct reliability was 0.9. The average variance extracted (AVE) for each construct exceeded 0.6. The six dimensions were in line with common criteria [38] and had good convergent validity (Fig. 1). Applying the criteria of Boomsma 2000 [39] and Byrne 2010 [40] produced a conclusion of good model fit (Table 6).

**Table 6:** Model fit index

Index of model fit	*χ*2	*χ*2/df	GFI	AGFI	RMSEA	SRMR	NNFI	IFI	CFI
Result of the study	3200.40 (*p* < 0.001)	3.89 (df = 696)	0.92	0.90	0.07(90 % CI = [0.06, 0.07])	0.06	0.86	0.91	0.91

GFI = Goodness of Fit Index

AGFI = Adjusted Goodness of Fit Index

RMSEA = Root Mean Square Error of Approximation

SRMR = Standardized Root Mean Square

NNFI = Non-Normed Fit Index

IFI = Incremental Fit Index

CFI = Comparative Fit Index

## Discussion

This study found that an outcomes-based framework for competencies in China would include diagnosis and management, communication, teamwork and administration, professional behavior, professional values, and technical procedural skills. We used the O*NET-GWA and O*NET-WS questionnaires and conducted quantitative analyses. In contrast, work done in other countries has relied mainly on qualitative methods, including theoretical analysis, behavioral event interviews, and the Delphi technique [10, 11]. It is reassuring that our work produced similar results.

The O*NET questionnaires were powerful tools for work analysis. The O*NET -Standard Occupational Classification system (O*NET-SOC) which included 1094 occupational titles have almost covered the entirety of occupations of US [41]. When applied the O*NET-GWA and O*NET-WS questionnaires to the occupation of doctor, the explained for the generic occupations needs to be kept close to the underlying elements of doctors. Thus the factors were named in light of medical content of the practice of physicians. The six-factor solution of the O*NET-GWA and O*NET-WS questionnaires was consistent with previous studies (Table 7) [25, 29] and outcome-based medical education reforms in China and worldwide. Compared with the competencies framework developed by the ACGME [42], our doctor’s competencies model was similar in diagnosis and management and technical procedural skills (the ACGME’s patient care and medical knowledge), communication, teamwork, and administration (the ACGME’s interpersonal and communication skills), and professional behavior and professional values (the ACGME’s professionalism). Likewise, compared with the CanMEDS framework [4], our doctor’s competencies model was similar in diagnosis and management and technical procedural skills (medical expert & scholar in CanMEDS), communication (communicator and health advocate in CanMEDS), teamwork and administration (collaborator and manager in CanMEDS), and professional behavior and professional values (professional in CanMEDS).

**Table 7:** Comparisons of competencies frameworks

Chinese doctor’s competencies framework	ACGME		CanMEDS		GMER	
Diagnosis and management	√	medical knowledge	√	medical expert	√	Clinical Skills
Technical procedural skills	√	patient care			√	Scientific Foundation of Medicine
Communication	√	interpersonal and communication skills	√	communicator;	√	Communication Skills
			√	health advocate	√	Management of Information
Teamwork and administration	√	Systems-based Practice	√	collaborator; manager	√	Population Health and Health Systems
Professional behavior	√	professionalism; Practice-based Learning and Improvement	√	professional; scholar	√	Professional Values, Attitudes, Behavior and Ethics; Critical Thinking and Research
Professional values						

Although these findings are consistent with frameworks developed elsewhere, our study does have limitations. We could only collect data from seven provinces. Although they cover different regions of the country, a broader sample might generate different results. Likewise, in our sample we had more urban than rural doctors. This might influence the applicability of our framework to the rural health system.

Since 1910, Abraham Flexner’s report has helped shape the face of medical education both in America and around the world [43]. In fact, China adopted a Flexnerian model as its medical institutions developed. However, the political, social, and economic environment has changed significantly since Flexner’s day [44]. This has generated the need for reform which has largely been filled by competencies-oriented medical education models [45] such as those developed in Canada [46] and the United States [47]. Our study provides a model, relevant to China, which will support educational reform efforts.

The findings of this study will have practical implications for health professions education. In the past, the majority of medical graduates in China have entered the health care system directly, without further training. Starting in 1993, China established postgraduate training programs for doctors [48]. However, clinical skills and medical knowledge are the primary focus of these training programs. Competencies such as communications, teamwork and administration, professional behavior, and professional values were not addressed. The findings of the study will help align the competencies developed during training with the needs of the healthcare system.

Finally, this study lays the groundwork for future research directions. Quantitative methods were used to establish this preliminary model of competencies. Further work based on qualitative methods such as behavioral event interview, focus group interviews, and the Delphi technique might suggest amendments to the model. In addition, collecting and analyzing the opinions of nurses, patients, and administrators will build and enrich the model of doctor’s competencies.

## Conclusions

Our results provide support for the reliability and validity of the Chinese version of the O*NET-GWS and O*NET-WS for doctors. The Chinese doctors’ competencies framework includes six elements: (a) technical procedural skills; (b) diagnosis and management; (c) teamwork and administration; (d) communication; (e) professional behavior; and (f) professional values. These findings are relevant to China, consistent with its current situation, and similar to those developed in other countries.

## Additional files

Additional file 1: **GWA questionnaire.** (DOC 2416 kb) Additional file 2: **WS questionnaire.** (DOC 283 kb)

## Abbreviations

O*NET-GWA: Occupational Information Network- General Work Activity
O*NET-WS: Occupational Information Network- Work Style

## References

[1] Grol R (2002). Changing Physicians’ Competence and Performance: Finding the Balance between the Individual and the Organization. J Contin Educ Health Prof.

[2] Epstein R, Hundert E (2002). Defining and assessing professional competence. JAMA internal medicine.

[3] Schwarz MR, Wojtczak A (2002). Global minimum essential requirements: a road towards competence-oriented medical education. Med Teach.

[4] Frank JR. The CanMEDS 2005 physician competency framework: Better standards, better physicians, better care. Royal College of Physicians and Surgeons of Canada. 2005.

[5] Council GM.How GMP applies to you. http://www.gmc-uk.org/guidance/good_medical_practice/how_gmp_applies_to_you.asp [Accessed 9 April 2013]

[6] Litzelman DK, Cottingham AH (2007). The new formal competency-based curriculum and informal curriculum at Indiana University School of Medicine: overview and five-year analysis. Acad Med.

[7] Ho MJ, Yu KH, Hirsh D, Huang TS, Yang PC (2011). Does one size fit all? Building a framework for medical professionalism. Acad Med.

[8] Al-Eraky MM, Chandratilake M (2012). How medical professionalism is conceptualised in Arabian context: a validation study. Med Teach.

[9] Frenk J, Chen L, Bhutta ZA, Cohen J, Crisp N, Evans T, et al. Health professionals for a new century: transforming education to strengthen health system in an interdependent world. Lancet. 2010;1923–1958. 10.1016/S0140-6736(10)61854-5 21112623

[10] Goligher EC, Ferguson ND, Kenny LP (2012). Core competency in mechanical ventilation: development of educational objectives using the Delphi technique. Crit Care Med.

[11] Magee T, Malloy CH (2011). A Delphi approach to developing a core competency framework for family practice registered nurses in Ontario, by Azadeh Moaveni, Anna Gallinaro, Lesley Gotlib Conn, Sheilagh Callahan, Melanie Hammond and Ivy Oandasan. Nurs leadersh.

[12] Larkin J, McDermott J, Simon DP, Simon HA (1980). Expert and novice performance in solving physics problems. Science.

[13] Johnson V (1998). Going from novice to expert. Revolution.

[14] DiBiase D, Corbin T, Fox T, Francica J, Green K, Jackson J (2010). The new geospatial technology competency model: Bringing workforce needs into focus. URISA Journal.

[15] Dierdorff EC, Wilson MA (2003). A meta-analysis of job analysis reliability. J Appl Psychol.

[16] Green ML, Aagaard EM, Caverzagie KJ, Chick DA, Holmboe E, Kane G (2009). Charting the road to competence: Developmental milestones for internal medicine residency training. J Grad Med Educ..

[17] DOL.Career Ladders and Lattices. http://www.careeronestop.org/competencymodel/careerpathway/CPWCllInstructions.aspx. [Accessed 8 July 2012]

[18] DOL.About O*NET. http://www.onetonline.org/. [Accessed 9 July 2012]

[19] Cifuentes M, Boyer J, Lombardi DA, Punnett L (2010). Use of O*NET as a job exposure matrix: A literature review. Am J Ind Med.

[20] Cifuentes M, Boyer J, Gore R, d'Errico A, Tessler J, Scollin P (2007). Inter-method agreement between O*NET and survey measures of psychosocial exposure among healthcare industry employees. Am J Ind Med.

[21] Clark CL. Factor Structures of the O* NET Occupational Descriptors. 2000.http://repository.lib.ncsu.edu/ir/handle/1840.16/640.

[22] Hadden WC, Kravets N, Muntaner C (2004). Descriptive dimensions of US occupations with data from the O*NET. Soc Sci Res.

[23] Peterson NG, Borman WC, Hanson MA, Kuabisiak UC (2009). Summary of results,implications for O*NET applicaitons, and future directions. In: *An occupational information system for the 21st century: the development of the O*NET.* edn.

[24] Jeanneret PR, Borman WC, Kubisiak C, Hanson MA (2009). General Work activity In: *An occupational information system for the 21st century: the development of the O*NET.* edn.

[25] LaPOLICE CC, Carter GW, Johnson JW (2008). Linking O* NET descriptors to occupational literacy requirements using job component validation. Pers Psychol.

[26] McLagan PA (1980). Competency models. Train Dev J.

[27] McConnell EA (2001). Competence vs. competency. Nurs Manage.

[28] Peterson NG, Mumford MD, Borman WC, Jeanneret PR, Flesishman EA (2009). Work styles. In: *An occupational information system for the 21st century: the development of the O*NET.* edn.

[29] Taylor PJ, LI WD, Shi K, Borman WC (2008). The transportability of job information across countries. Pers Psychol.

[30] National Health and Family Planning Commission of the PRC (2012). *China Health Statistical Yearbook 2011*. http://www.nhfpc.gov.cn. [Accessed 2012, Sep 15]

[31] 31. CMB-CMU-FAIMER.About NCC. http://cmbcmu.faimerfri.org/information-on-ncc/ [Accessed 2012 May,3]

[32] Ji Z, Yongwen C, Yuanfeng H (1995). Natural geography of China.

[33] Ferro MA, Avison WR, Campbell MK, Speechley KN (2011). The impact of maternal depressive symptoms on health-related quality of life in children with epilepsy: a prospective study of family environment as mediators and moderators. Epilepsia.

[34] Kaiser HF (1960). The application of electronic computers to factor analysis. Educ Psychol Meas.

[35] Cattell RB (1966). The scree test for the number of factors. Multivar Behav Res.

[36] Cronbach LJ (1951). Coefficient alpha and the internal structure of tests. Psychometrika.

[37] Brown TA. Confirmatory factor analysis for applied research. Guilford Press. 2006.

[38] Fornell C, Larcker DF. Evaluating structural equation models with unobservable variables and measurement error. J mark res. 1981;39–50.

[39] Boomsma A (2000). Reporting analyses of covariance structures. Struct equ model.

[40] Byrne BM. Structural equation modeling with AMOS: Basic concepts, applications, and programming. CRC Press. 2009.

[41] Levine J, Nottingham J, Paige B, Lewis P (2000). Transitioning O* NET to the Standard Occupational Classification.

[42] Caverzagie KJ, Iobst WF, Aagaard EM, Hood S, Chick DA, Kane GC (2013). The internal medicine reporting milestones and the next accreditation system. Ann Intern Med.

[43] Flexner A (1910). Medical education in the United States and Canada: a report to the Carnegie Foundation for the Advancement of Teaching.

[44] Molly Cooke DMI, Sullivan W (2006). American Medical Education 100 Years after the Flexner Report. N Engl J Med.

[45] Lowry BN, Vansaghi LM, Rigler SK, Stites SW (2013). Applying the Milestones in an Internal Medicine Residency Program Curriculum: A Foundation for Outcomes-Based Learner Assessment Under the Next Accreditation System. Acad Med.

[46] Michels NR, Denekens J, Driessen EW, Van Gaal LF, Bossaert LL, De Winter BY (2012). A Delphi study to construct a CanMEDS competence based inventory applicable for workplace assessment. BMC Med Educ.

[47] Nasca TJ, Philibert I, Brigham T, Flynn TC (2012). The next GME accreditation system--rationale and benefits. N Engl J Med.

[48] MOH.Trial implementation of standardized training program for residents. http://www.nhfpc.gov.cn [Accessed 2012 May,3]

