# Supplementary material for: Identifying the competencies of doctors in China
Source: BMC Med Educ. 2015 Nov 25;15:207. doi: 10.1186/s12909-015-0495-y (PMC4659240; doi:10.1186/s12909-015-0495-y)
Supplement: Additional file 1: — GWA questionnaire. (DOC 2416 kb) [file 12909_2015_495_MOESM1_ESM.doc]

**Instructions for Making General Work Activities Ratings**

These questions are about work activities. A *work activity* is a set of similar actions that are performed together in many different jobs. You will be asked about a series of different work activities and how they relate to *your current job* - that is, the job you hold now.

**Each activity in this questionnaire is named and defined.**

For example:

| **Getting Information** | **Observing, receiving, and otherwise obtaining information from all relevant sources.** |
| --- | --- |

You are then asked to answer two questions about that activity:

***A***

***How important is the activity to your current job?***

For example:

**How important is GETTING INFORMATION to the performance of *your current job*?**

Mark your answer by putting an **X** through the number that represents your answer.

Do not mark on the line between the numbers.

***If you rate the activity as Not Important to the performance of your job, mark the one [ ] then skip over question B and proceed to the next activity.**

***B***

***What level of the activity is needed to perform your current job?***

To help you understand what we mean by **level**, we provide you with examples of job-related activities at different levels. For example:

**What level of GETTING INFORMATION is needed to perform *your current job*?**

Mark your answer by putting an **X** through the number that represents your answer.

Do not mark on the line between the numbers.

| 1. **Getting Information** | **Observing, receiving, and otherwise obtaining information from all relevant sources.** |
| --- | --- |

**A. How important is GETTING INFORMATION to the performance of *your current job*?**

* If you marked Not Important, skip LEVEL below and go on to the next activity.

**B. What level of GETTING INFORMATION is needed to perform *your current job*?**

| 1. **Identifying Objects, Actions, and Events** | **Identifying information by categorizing, estimating, recognizing differences or similarities, and detecting changes in circumstances or events.** |
| --- | --- |

**A. How important is IDENTIFYING OBJECTS, ACTIONS, AND EVENTS to the performance of *your current job*?**

* If you marked Not Important, skip LEVEL below and go on to the next activity.

**B. What level of IDENTIFYING OBJECTS, ACTIONS, AND EVENTS is needed to perform *your current job*?**

| **3. Monitoring Processes, Materials, or Surroundings** | **Monitoring and reviewing information from materials, events, or the environment to detect or assess problems.** |
| --- | --- |

**A. How important is MONITORING PROCESSES, MATERIALS, OR SURROUNDINGS to the performance of *your current job*?**

* If you marked Not Important, skip LEVEL below and go on to the next activity.

**B. What level of MONITORING PROCESSES, MATERIALS, OR SURROUNDINGS is needed to perform *your current job*?**

| **4. Inspecting Equipment, Structures, or Materials** | **Inspecting equipment, structures, or materials to identify the cause of errors or other problems or defects.** |
| --- | --- |

**A. How important is INSPECTING EQUIPMENT, STRUCTURES, OR MATERIALS to the performance of *your current job*?**

* If you marked Not Important, skip LEVEL below and go on to the next activity.

**B. What level of INSPECTING EQUIPMENT, STRUCTURES, OR MATERIALS is needed to perform *your current job*?**

| **5. Estimating the Quantifiable Characteristics of Products, Events, or Information** | **Estimating sizes, distances, and quantities; or determining time, costs, resources, or materials needed to perform a work activity.** |
| --- | --- |

**A. How important is ESTIMATING THE QUANTIFIABLE CHARACTERISTICS OF PRODUCTS, EVENTS, OR INFORMATION to the performance of *your current job*?**

* If you marked Not Important, skip LEVEL below and go on to the next activity.

**B. What level of ESTIMATING THE QUANTIFIABLE CHARACTERISTICS OF PRODUCTS, EVENTS, OR INFORMATION is needed to perform *your current job*?**

| **6. Judging the Qualities of Objects, Services, or People** | **Assessing the value, importance, or quality of things or people.** |
| --- | --- |

**A. How important is JUDGING THE QUALITIES OF OBJECTS, SERVICES, OR PEOPLE to the performance of *your current job*?**

* If you marked Not Important, skip LEVEL below and go on to the next activity.

**B. What level of JUDGING THE QUALITIES OF OBJECTS, SERVICES, OR PEOPLE is needed to perform *your current job*?**

| **7. Evaluating Information to Determine Compliance with Standards** | **Using relevant information and individual judgment to determine whether events or processes comply with laws, regulations, or standards.** |
| --- | --- |

**A. How important is EVALUATING INFORMATION TO DETERMINE COMPLIANCE WITH STANDARDS to the performance of *your current job*?**

* If you marked Not Important, skip LEVEL below and go on to the next activity.

**B. What level of EVALUATING INFORMATION TO DETERMINE COMPLIANCE WITH STANDARDS is needed to perform *your current job*?**

| 1. **Processing Information** | **Compiling, coding, categorizing, calculating, tabulating, auditing, or verifying information or data.** |
| --- | --- |

**A. How important is PROCESSING INFORMATION to the performance of *your current job*?**

* If you marked Not Important, skip LEVEL below and go on to the next activity.

**B. What level of PROCESSING INFORMATION is needed to perform *your current job*?**

| 1. **Analyzing Data or Information** | **Identifying the underlying principles, reasons, or facts of information by breaking down information or data into separate parts.** |
| --- | --- |

**A. How important is ANALYZING DATA OR INFORMATION to the performance of *your current job*?**

* If you marked Not Important, skip LEVEL below and go on to the next activity.

**B. What level of ANALYZING DATA OR INFORMATION is needed to perform *your current job*?**

| **10. Making Decisions and Solving Problems** | **Analyzing information and evaluating results to choose the best solution and solve problems.** |
| --- | --- |

**A. How important is MAKING DECISIONS AND SOLVING PROBLEMS to the performance of *your current job*?**

* If you marked Not Important, skip LEVEL below and go on to the next activity.

**B. What level of MAKING DECISIONS AND SOLVING PROBLEMS is needed to perform *your current job*?**

| 1. **Thinking Creatively** | **Developing, designing, or creating new applications, ideas, relationships, systems, or products, including artistic contributions.** |
| --- | --- |

**A. How important is THINKING CREATIVELY to the performance of *your current job*?**

* If you marked Not Important, skip LEVEL below and go on to the next activity.

**B. What level of THINKING CREATIVELY is needed to perform *your current job*?**

| 1. **Updating and Using Relevant Knowledge** | **Keeping up-to-date technically and applying new knowledge to your job.** |
| --- | --- |

**A. How important is UPDATING AND USING RELEVANT KNOWLEDGE to the performance of *your current job*?**

* If you marked Not Important, skip LEVEL below and go on to the next activity.

**B. What level of UPDATING AND USING RELEVANT KNOWLEDGE is needed to perform *your current job*?**

| 1. **Developing Objectives and Strategies** | **Establishing long-range objectives and specifying the strategies and actions to achieve them.** |
| --- | --- |

**A. How important is DEVELOPING OBJECTIVES AND STRATEGIES to the performance of *your current job*?**

* If you marked Not Important, skip LEVEL below and go on to the next activity.

**B. What level of DEVELOPING OBJECTIVES AND STRATEGIES is needed to perform *your current job*?**

| 1. **Scheduling Work and Activities** | **Scheduling events, programs, and activities, as well as the work of others.** |
| --- | --- |

**A. How important is SCHEDULING WORK AND ACTIVITIES to the performance of *your current job*?**

* If you marked Not Important, skip LEVEL below and go on to the next activity.

**B. What level of SCHEDULING WORK AND ACTIVITIES is needed to perform *your current job*?**

| 1. **Organizing, Planning, and Prioritizing Work** | **Developing specific goals and plans to prioritize, organize, and accomplish your work.** |
| --- | --- |

**A. How important is ORGANIZING, PLANNING, AND PRIORITIZING WORK to the performance of *your current job*?**

* If you marked Not Important, skip LEVEL below and go on to the next activity.

**B. What level of ORGANIZING, PLANNING, AND PRIORITIZING WORK is needed to perform *your current job*?**

| 1. **Performing General Physical Activities** | **Performing physical activities that require considerable use of your arms and legs and moving your whole body, such as climbing, lifting, balancing, walking, stooping, and handling materials.** |
| --- | --- |

**A. How important is PERFORMING GENERAL PHYSICAL ACTIVITIES to the performance of *your current job*?**

* If you marked Not Important, skip LEVEL below and go on to the next activity.

**B. What level of PERFORMING GENERAL PHYSICAL ACTIVITIES is needed to perform *your current job*?**

| 1. **Handling and Moving Objects** | **Using hands and arms in handling, installing, positioning, and moving materials, and manipulating things.** |
| --- | --- |

**A. How important is HANDLING AND MOVING OBJECTS to the performance of *your current job*?**

* If you marked Not Important, skip LEVEL below and go on to the next activity.

**B. What level of HANDLING AND MOVING OBJECTS is needed to perform *your current job*?**

| 1. **Controlling Machines and Processes** | **Using either control mechanisms or direct physical activity to operate machines or processes (not including computers or vehicles).** |
| --- | --- |

**A. How important is CONTROLLING MACHINES AND PROCESSES to the performance of *your current job*?**

* If you marked Not Important, skip LEVEL below and go on to the next activity.

**B. What level of CONTROLLING MACHINES AND PROCESSES is needed to perform *your current job*?**

| 1. **Working with Computers** | **Using computers and computer systems (including hardware and software) to program, write software, set up functions, enter data, or process information.** |
| --- | --- |

**A. How important is WORKING WITH COMPUTERS to the performance of *your current job*?**

* If you marked Not Important, skip LEVEL below and go on to the next activity.

**B. What level of WORKING WITH COMPUTERS is needed to perform *your current job*?**

| **20. Operating Vehicles, Mechanized Devices, or Equipment** | **Running, maneuvering, navigating, or driving vehicles or mechanized equipment, such as forklifts, passenger vehicles, aircraft, or water craft.** |
| --- | --- |

**A. How important is OPERATING VEHICLES, MECHANIZED DEVICES, OR EQUIPMENT to the performance of *your current job*?**

* If you marked Not Important, skip LEVEL below and go on to the next activity.

**B. What level of OPERATING VEHICLES, MECHANIZED DEVICES, OR EQUIPMENT is needed to perform *your current job*?**

| **21. Drafting, Laying Out, and Specifying Technical Devices, Parts, and Equipment** | **Providing documentation, detailed instructions, drawings, or specifications to tell others about how devices, parts, equipment, or structures are to be fabricated, constructed, assembled, modified, maintained, or used.** |
| --- | --- |

**A. How important is DRAFTING, LAYING OUT, AND SPECIFYING TECHNICAL DEVICES, PARTS, AND EQUIPMENT to the performance of *your current job*?**

* If you marked Not Important, skip LEVEL below and go on to the next activity.

**B. What level of DRAFTING, LAYING OUT, AND SPECIFYING TECHNICAL DEVICES, PARTS, AND EQUIPMENT is needed to perform *your current job*?**

| 1. **Repairing and Maintaining Mechanical Equipment** | **Servicing, repairing, adjusting, and testing machines, devices, moving parts, and equipment that operate primarily on the basis of mechanical (not electronic) principles.** |
| --- | --- |

**A. How important is REPAIRING AND MAINTAINING MECHANICAL EQUIPMENT to the performance of *your current job*?**

* If you marked Not Important, skip LEVEL below and go on to the next activity.

**B. What level of REPAIRING AND MAINTAINING MECHANICAL EQUIPMENT is needed to perform *your current job*?**

| 1. **Repairing and Maintaining Electronic Equipment** | **Servicing, repairing, calibrating, regulating, fine-tuning, or testing machines, devices, and equipment that operate primarily on the basis of electrical or electronic (not mechanical) principles.** |
| --- | --- |

**A. How important is REPAIRING AND MAINTAINING ELECTRONIC EQUIPMENT to the performance of *your current job*?**

* If you marked Not Important, skip LEVEL below and go on to the next activity.

**B. What level of REPAIRING AND MAINTAINING ELECTRONIC EQUIPMENT is needed to perform *your current job*?**

| 1. **Documenting/Recording Information** | **Entering, transcribing, recording, storing, or maintaining information in written or electronic/magnetic form.** |
| --- | --- |

**A. How important is DOCUMENTING/RECORDING INFORMATION to the performance of *your current job*?**

* If you marked Not Important, skip LEVEL below and go on to the next activity.

**B. What level of DOCUMENTING/RECORDING INFORMATION is needed to perform *your current job*?**

| **25. Interpreting the Meaning of Information for Others** | **Translating or explaining what information means and how it can be used.** |
| --- | --- |

**A. How important is INTERPRETING THE MEANING OF INFORMATION FOR OTHERS to the performance of *your current job*?**

* If you marked Not Important, skip LEVEL below and go on to the next activity.

**B. What level of INTERPRETING THE MEANING OF INFORMATION FOR OTHERS is needed to perform *your current job*?**

| **26. Communicating with Supervisors, Peers, or Subordinates** | **Providing information to supervisors, coworkers, and subordinates by telephone, in written form, e-mail, or in person.** |
| --- | --- |

**A. How important is COMMUNICATING WITH SUPERVISORS, PEERS, OR SUBORDINATES to the performance of *your current job*?**

* If you marked Not Important, skip LEVEL below and go on to the next activity.

**B. What level of COMMUNICATING WITH SUPERVISORS, PEERS, OR SUBORDINATES is needed to perform *your current job*?**

| **27. Communicating with People Outside the Organization** | **Communicating with people outside the organization, representing the organization to customers, the public, government, and other external sources. This information can be exchanged in person, in writing, or by telephone or e-mail.** |
| --- | --- |

**A. How important is COMMUNICATING WITH PEOPLE OUTSIDE THE ORGANIZATION to the performance of *your current job*?**

* If you marked Not Important, skip LEVEL below and go on to the next activity.

**B. What level of COMMUNICATING WITH PEOPLE OUTSIDE THE ORGANIZATION is needed to perform *your current job*?**

| **28. Establishing and Maintaining Interpersonal Relationships** | **Developing constructive and cooperative working relationships with others and maintaining them over time.** |
| --- | --- |

**A. How important is ESTABLISHING AND MAINTAINING INTERPERSONAL RELATIONSHIPS to the performance of *your current job*?**

* If you marked Not Important, skip LEVEL below and go on to the next activity.

**B. What level of ESTABLISHING AND MAINTAINING INTERPERSONAL RELATIONSHIPS is needed to perform *your current job*?**

| 1. **Assisting and Caring for Others** | **Providing personal assistance, medical attention, emotional support, or other personal care to others such as coworkers, customers, or patients.** |
| --- | --- |

**A. How important is ASSISTING AND CARING FOR OTHERS to the performance of *your current job*?**

* If you marked Not Important, skip LEVEL below and go on to the next activity.

**B. What level of ASSISTING AND CARING FOR OTHERS is needed to perform *your current job*?**

| 1. **Selling or Influencing Others** | **Convincing others to buy merchandise/goods or to otherwise change their minds or actions.** |
| --- | --- |

**A. How important is SELLING OR INFLUENCING OTHERS to the performance of *your current job*?**

* If you marked Not Important, skip LEVEL below and go on to the next activity.

**B. What level of SELLING OR INFLUENCING OTHERS is needed to perform *your current job*?**

| 1. **Resolving Conflicts and Negotiating with Others** | **Handling complaints, settling disputes, and resolving grievances and conflicts, or otherwise negotiating with others.** |
| --- | --- |

**A. How important is RESOLVING CONFLICTS AND NEGOTIATING WITH OTHERS to the performance of *your current job*?**

* If you marked Not Important, skip LEVEL below and go on to the next activity.

**B. What level of RESOLVING CONFLICTS AND NEGOTIATING WITH OTHERS is needed to perform *your current job*?**

| **32. Performing for or Working Directly with the Public** | **Performing for people or dealing directly with the public. This includes serving customers in restaurants and stores, and receiving clients or guests.** |
| --- | --- |

**A. How important is PERFORMING FOR OR WORKING DIRECTLY WITH THE PUBLIC to the performance of *your current job*?**

* If you marked Not Important, skip LEVEL below and go on to the next activity.

**B. What level of PERFORMING FOR OR WORKING DIRECTLY WITH THE PUBLIC is needed to perform *your current job*?**

| 1. **Coordinating the Work and Activities of Others** | **Getting members of a group to work together to accomplish tasks.** |
| --- | --- |

**A. How important is COORDINATING THE WORK AND ACTIVITIES OF OTHERS to the performance of *your current job*?**

* If you marked Not Important, skip LEVEL below and go on to the next activity.

**B. What level of COORDINATING THE WORK AND ACTIVITIES OF OTHERS is needed to perform *your current job*?**

| 1. **Developing and Building Teams** | **Encouraging and building mutual trust, respect, and cooperation among team members.** |
| --- | --- |

**A. How important is DEVELOPING AND BUILDING TEAMS to the performance of *your current job*?**

* If you marked Not Important, skip LEVEL below and go on to the next activity.

**B. What level of DEVELOPING AND BUILDING TEAMS is needed to perform *your current job*?**

| 1. **Training and Teaching Others** | **Identifying the educational needs of others, developing formal educational or training programs or classes, and teaching or instructing others.** |
| --- | --- |

**A. How important is TRAINING AND TEACHING OTHERS to the performance of *your current job*?**

* If you marked Not Important, skip LEVEL below and go on to the next activity.

**B. What level of TRAINING AND TEACHING OTHERS is needed to perform *your current job*?**

| 1. **Guiding, Directing, and Motivating Subordinates** | **Providing guidance and direction to subordinates, including setting performance standards and monitoring performance.** |
| --- | --- |

**A. How important is GUIDING, DIRECTING, AND MOTIVATING SUBORDINATES to the performance of *your current job*?**

* If you marked Not Important, skip LEVEL below and go on to the next activity.

**B. What level of GUIDING, DIRECTING, AND MOTIVATING SUBORDINATES is needed to perform *your current job*?**

| 1. **Coaching and Developing Others** | **Identifying the developmental needs of others and coaching, mentoring, or otherwise helping others to improve their knowledge or skills.** |
| --- | --- |

**A. How important is COACHING AND DEVELOPING OTHERS to the performance of *your current job*?**

* If you marked Not Important, skip LEVEL below and go on to the next activity.

**B. What level of COACHING AND DEVELOPING OTHERS is needed to perform *your current job*?**

| 1. **Providing Consultation and Advice to Others** | **Providing guidance and expert advice to management or other groups on technical, systems-, or process-related topics.** |
| --- | --- |

**A. How important is PROVIDING CONSULTATION AND ADVICE TO OTHERS to the performance of *your current job*?**

* If you marked Not Important, skip LEVEL below and go on to the next activity.

**B. What level of PROVIDING CONSULTATION AND ADVICE TO OTHERS is needed to perform *your current job*?**

| **39. Performing Administrative Activities** | **Performing day-to-day administrative tasks such as maintaining information files and processing paperwork.** |
| --- | --- |

**A. How important is PERFORMING ADMINISTRATIVE ACTIVITIES to the performance of *your current job*?**

* If you marked Not Important, skip LEVEL below and go on to the next activity.

**B. What level of PERFORMING ADMINISTRATIVE ACTIVITIES is needed to perform *your current job*?**

| **40. Staffing Organizational Units** | **Recruiting, interviewing, selecting, hiring, and promoting employees in an organization.** |
| --- | --- |

**A. How important is STAFFING ORGANIZATIONAL UNITS to the performance of *your current job*?**

* If you marked Not Important, skip LEVEL below and go on to the next activity.

**B. What level of STAFFING ORGANIZATIONAL UNITS is needed to perform *your current job*?**

| 1. **Monitoring and Controlling Resources** | **Monitoring and controlling resources and overseeing the spending of money.** |
| --- | --- |

**A. How important is MONITORING AND CONTROLLING RESOURCES to the performance of *your current job*?**

* If you marked Not Important, skip LEVEL below and go on to the next activity.

**B. What level of MONITORING AND CONTROLLING RESOURCES is needed to perform *your current job*?**
