# Supplementary material for: Identifying the competencies of doctors in China
Source: BMC Med Educ. 2015 Nov 25;15:207. doi: 10.1186/s12909-015-0495-y (PMC4659240; doi:10.1186/s12909-015-0495-y)
Supplement: Additional file 2: — WS questionnaire. (DOC 283 kb) [file 12909_2015_495_MOESM2_ESM.doc]

**Instructions for Making Work Style Ratings**

These questions are about work styles. *A* ***Work Style*** *is a personal characteristic that can affect how well someone does a job.* You will be asked about a series of different work styles and how they relate to *your current job* – that is, the job you hold now.

First, each work style is named and defined. For example:

| **Stress Tolerance** | **Job requires accepting criticism and dealing calmly and effectively with high-stress situations.** |
| --- | --- |

Then you are asked *How important is this characteristic to the performance of your current job?* For example:

**How important is STRESS TOLERANCE to the performance of *your current job*?**

Mark your answer by putting an **X** through the number that represents your answer.

Do not mark on the line between the numbers.

| **1. Achievement/Effort** | **Job requires establishing and maintaining personally challenging achievement goals and exerting effort toward mastering tasks.** |
| --- | --- |

**How important is ACHIEVEMENT/EFFORT to the performance of *your current job*?**

| **2. Persistence** | **Job requires persistence in the face of obstacles.** |
| --- | --- |

**How important is PERSISTENCE** **to the performance of *your current job*?**

| **3. Initiative** | **Job requires a willingness to take on responsibilities and challenges.** |
| --- | --- |

**How important is INITIATIVE** **to the performance of *your current job*?**

| **4. Leadership** | **Job requires a willingness to lead, take charge, and offer opinions and direction.** |
| --- | --- |

**How important is LEADERSHIP** **to the performance of *your current job*?**

| **5. Cooperation** | **Job requires being pleasant with others on the job and displaying a good-natured, cooperative attitude.** |
| --- | --- |

**How important is COOPERATION** **to the performance of *your current job*?**

| **6. Concern for Others** | **Job requires being sensitive to others' needs and feelings, and being understanding and helpful to others on the job.** |
| --- | --- |

**How important is CONCERN FOR OTHERS** **to the performance of *your current job*?**

| **7. Social Orientation** | **Job requires preferring to work with others rather than alone, and being personally connected with others on the job.** |
| --- | --- |

**How important is SOCIAL ORIENTATION to the performance of *your current job*?**

| **8. Self-Control** | **Job requires maintaining composure, keeping emotions in check, controlling anger, and avoiding aggressive behavior, even in very difficult situations.** |
| --- | --- |

**How important is SELF-CONTROL** **to the performance of *your current job*?**

| **9. Stress Tolerance** | **Job requires accepting criticism and dealing calmly and effectively with high-stress situations.** |
| --- | --- |

**How important is STRESS TOLERANCE** **to the performance of *your current job*?**

| **10. Adaptability/Flexibility** | **Job requires being open to change (positive or negative) and to considerable variety in the workplace.** |
| --- | --- |

**How important is ADAPTABILITY/FLEXIBILITY** **to the performance of *your current job*?**

| **11. Dependability** | **Job requires being reliable, responsible, and dependable, and fulfilling obligations.** |
| --- | --- |

**How important is DEPENDABILITY** **to the performance of *your current job*?**

| **12. Attention to Detail** | **Job requires being careful about details and thorough in completing tasks.** |
| --- | --- |

**How important is ATTENTION TO DETAIL** **to the performance of *your current job*?**

| **13. Integrity** | **Job requires being honest and ethical.** |
| --- | --- |

**How important is INTEGRITY** **to the performance of *your current job*?**

| **14. Independence** | **Job requires developing one's own ways of doing things, guiding oneself with little or no supervision, and depending on oneself to get things done.** |
| --- | --- |

**How important is INDEPENDENCE** **to the performance of *your current job*?**

| **15. Innovation** | **Job requires creativity and alternative thinking to develop new ideas for and answers to work-related problems.** |
| --- | --- |

**How important is INNOVATION** **to the performance of *your current job*?**

| **16. Analytical Thinking** | **Job requires analyzing information and using logic to address work-related issues and problems.** |
| --- | --- |

**How important is ANALYTICAL THINKING** **to the performance of *your current job*?**
